# Impact of regular additional endobiliary radiofrequency ablation on survival of patients with advanced extrahepatic cholangiocarcinoma under systemic chemotherapy

**DOI:** 10.1038/s41598-021-04297-2

**Published:** 2022-01-19

**Authors:** Maria A. Gonzalez-Carmona, Christian Möhring, Robert Mahn, Taotao Zhou, Alexandra Bartels, Farsaneh Sadeghlar, Maximilian Bolch, Annabelle Vogt, Dominik J. Kaczmarek, Dominik J. Heling, Leona Dold, Jacob Nattermann, Vittorio Branchi, Hanno Matthaei, Steffen Manekeller, Jörg C. Kalff, Christian P. Strassburg, Raphael U. Mohr, Tobias J. Weismüller

**Affiliations:** 1 Department of Internal Medicine I, University Hospital of Bonn, Venusberg-Campus 1, 53127 Bonn, Germany; 2 Department of Surgery, University Hospital of Bonn, Bonn, Germany

**Keywords:** Cancer, Gastroenterology, Oncology

## Abstract

Prognosis of patients with advanced extrahepatic cholangiocarcinoma (eCCA) is poor. The current standard first-line treatment is systemic chemotherapy (CT) with gemcitabine and a platinum derivate. Additionally, endobiliary radiofrequency ablation (eRFA) can be applied to treat biliary obstructions. This study aimed to evaluate the additional benefit of scheduled regular eRFA in a real-life patient cohort with advanced extrahepatic cholangiocarcinoma under standard systemic CT. All patients with irresectable eCCA treated at University Hospital Bonn between 2010 and 2020 were eligible for inclusion. Patients were stratified according to treatment: standard CT (n = 26) vs. combination of eRFA with standard CT (n = 40). Overall survival (OS), progression free survival (PFS), feasibility and toxicity were retrospectively analyzed using univariate and multivariate approaches. Combined eRFA and CT resulted in significantly longer median OS (17.3 vs. 8.6 months, p = 0.004) and PFS (12.9 vs. 5.7 months, p = 0.045) compared to the CT only group. While groups did not differ regarding age, sex, tumor stage and chemotherapy treatment regimen, mean MELD was even higher (10.1 vs. 6.7, p = 0.015) in the eRFA + CT group. The survival benefit of concomitant eRFA was more evident in the subgroup with locally advanced tumors. Severe hematological toxicities (CTCAE grades 3 – 5) did not differ significantly between the groups. However, therapy-related cholangitis occurred more often in the combined treatment group (p = 0.031). Combination of eRFA and systemic CT was feasible, well-tolerated and could significantly prolong survival compared to standard CT alone. Thus, eRFA should be considered during therapeutic decision making in advanced eCCA.

## Introduction

Biliary tract cancer, representing 3% of all gastrointestinal malignancies, is a rare disease with an incidence of 2–3/100,000 in the Western world^1–3^. The only curative treatment is radical surgery, but due to a locally advanced or metastatic stage most patients are eligible for palliative therapies only^4^. Despite the suggested survival benefits in the randomized phase III BILCAP trial by adjuvant administration of capecitabine for resected intrahepatic cholangiocarcinoma, high rates of disease recurrence are still contributing to a poor overall prognosis^5–7^.The pivotal phase III ABC-02 trial established the current palliative systemic first-line chemotherapy (CT) standard with gemcitabine and cisplatin^8^. A large number of trials investigating other combined chemotherapies or the addition of a third agent to gemcitabine and cisplatin (e.g., nab-paclitaxel, S1) failed to improve survival benefit to gemcitabine plus platinum derivate^9^. In 2021, pemigatinib, the first targeted therapy for patients with unresectable cholangiocarcinoma previously treated with fibroblast growth factor receptor 2 (FGFR2) fusion or rearrangement has been approved based on the results of the phase II FIGHT-202 trial^10^. Further trials using checkpoint inhibitors and other targeted therapies (e.g. pembrolizumab, nivolumab, anlotinib) are ongoing and results are eagerly awaited^9^.

In eCCA, concomitant endoscopic placement of biliary metal or plastic stents is an established procedure to ensure biliary drainage and to reduce the risk of obstructive cholangitis^11^. To improve local tumor control and biliary strictures, local ablative therapies, such as endobiliary radiofrequency ablation (eRFA) or photodynamic therapy (PDT), are applied individually.

eRFA uses a high frequency alternating current applied via a bipolar probe to generate heat that induces localized tissue necrosis^12,13^. Similarly, in patients with small intrahepatic cholangiocarcinoma (iCCA), percutaneous thermal ablation through RFA or microwave ablation has been shown to be safe and effective in terms of survival^14^. Studies have also supplied evidence that eRFA prolongs stent patency in cases of eCCA, which may be beneficial in improving survival^15–17^. However, available evidence remains insufficient, as it is mainly derived from retrospective studies with a limited number of patients with malignant biliary obstruction of diverse etiology. Some data is available for eRFA in the setting of eCCA^18–20^. To the best of our knowledge, only one study has evaluated the efficacy of eRFA in eCCA limited to Bismuth type I and II and distal cholangiocarcinoma using a prospective cohort design. Yang et al. reported a significantly longer overall survival (OS) in the eRFA + stent group compared to the stent-only group (13.2 ± 0.6 vs. 8.3 ± 0.5 months; p < 0.001)^21^. However, patients receiving CT were excluded, hence data evaluating possible synergism of eRFA in combination with current standard of care CT are lacking. Thus, the aim of this study was to evaluate the benefit of concomitant eRFA in combination with systemic CT compared to CT alone in a real-life cohort of patients with advanced eCCA.

## Materials and methods

### Patient population

**Figure 1:**
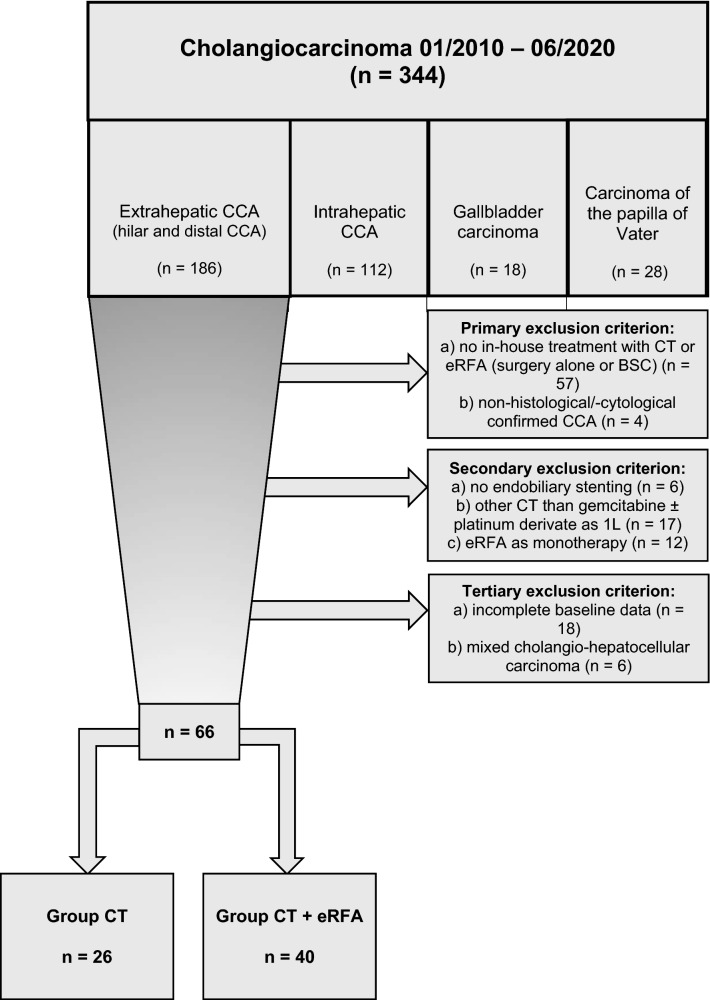
Flow-chart of patients. *1L* first-line, *BSC* best supportive care, *CCA* cholangiocarcinoma, *CT* chemotherapy, *eRFA* endobiliary radiofrequency ablation, *n* number.

All patients diagnosed with non-curative resectable biopsy-proven eCCA between 2010 and 2020 at the University Hospital of Bonn, Germany, who received palliative systemic first-line CT with gemcitabine ± platinum derivate and who were treated with endobiliary stenting were eligible for inclusion (Fig. 1). Patients were stratified according to treatment: combination eRFA + CT (n = 40) or standard CT only (n = 26). Diagnosis was based on histological (n = 64) or cytological (n = 2) validation. Patients were considered inoperable because of advanced stage of disease (vascular invasion corresponding T4 stage of TNM classification or distant metastasis corresponding N2 and/or M1 stages of TNM classification) or poor performance status due to relevant comorbidities. Patients were treated with systemic CT if performance status, hepatic and renal function were considered sufficient. Concomitant eRFA was offered to every patient with obstructive biliary symptoms and informed consent was obtained. Therapy decisions were made following consensus decision by our interdisciplinary tumor board and in agreement with the individual patient wishes, especially considering toxicities of CT.

### Therapeutic procedures

As first^-^line standard CT, a combination of gemcitabine (1000 mg/m^2^) and cisplatin (25 mg/m^2^) was applied. Unfit patients were offered gemcitabine monotherapy and, in case of renal impairment, cisplatin was replaced by oxaliplatin (80 mg/m^2^). Second-line therapies with FOLFIRI (folinic acid, fluorouracil and irinotecan), capecitabine or cetuximab were applied in 16.6% of patients.

Bile duct stenting was performed via endoscopic retrograde cholangiography (ERC) to treat and prevent cholestasis. Plastic stents (7Fr or 10Fr double-pigtail-stents, ENDO-FLEX, Voerde, Germany) were routinely replaced after 8–12 weeks or earlier in case of cholangitis or progressive cholestasis. When anatomically feasible, self-expanding metal stents (covered or uncovered 10 mm Wallstent™, Boston Scientific, Marlborough, MA, USA) were applied in case of recurrent early dysfunction of plastic stents or if patient performance did not allow scheduled stent replacements. In four patients (eRFA + CT: 2, CT: 2), bile duct stenting via ERC was not possible and cholestasis was treated with percutaneous transhepatic cholangiodrainage (PTCD). A further 11 patients received PTCD during follow-up due to altered anatomy following surgery or disease progression (Table 2). In 38 patients (95%), eRFA was performed through ERC and in two patients (5%), percutaneously. After removal of plastic stents and debris, the 8Fr RFA probe (Habib EndoHPB Bipolar Radiofrequency Catheter, Boston Scientific, Marlborough, MA, USA) was placed into the strictured duct using a guidewire. Cylindrical ablation over a length of 25 mm was performed for 90 s (VIO 200, Soft Coag mode, effect 8, 10 W, ERBE, Tübingen, Germany). The electrode was allowed to cool down for 60 s before being moved. Stepwise ablation from proximal to distal was performed in strictures longer than 25 mm. After eRFA, plastic stents were inserted to ensure adequate decompression of the stricture and bile drainage. If feasible, eRFA was repeated every 3–4 months.

### Data collection and study design

This is a single institution retrospective analysis. Baseline parameters (Table 1) were recorded prior to therapy. Patients were followed until death or end of observation period in May 2020. Patients lost to follow-up were censored at date of last visit. Tumor response was assessed by computer tomography and/or magnetic resonance imaging, which were performed regularly every 2–3 months. CT toxicity was recorded according to the common terminology criteria for adverse events version 4.03 (CTCAE) for grades 3–5. Median OS (mOS) was defined as the time range from application of first tumor-specific therapy until death. Median progression free survival (PFS) was defined as the time range from first tumor-specific therapy until progressive disease or death.

**Table 1:** Baseline characteristics.

Parameters	eRFA + CT (n = 40)	CT (n = 26)	P-value
Age [years]	69.0 (57.5; 76.8)	66.5 (57.0; 72.0)	0.187
**Gender**			0.522
Male	23 (57.5)	17 (65.4)	
Female	17 (42.5)	9 (34.6)	
**Tumor localization**			0.682
Bismuth 1–2 and distal CCA	9 (22.5)	7 (26.9)	
Bismuth 3–4	31 (77.5)	19 (73.1)	
**M status**			0.315
M0	25 (62.5)	13 (50.0)	
M1	15 (37.5)	13 (50.0)	
**Grading**			0.357
G1	8 (20.0)	2 (7.7)	
G2	17 (42.5)	9 (34.6)	
G3	9 (22.5)	9 (34.6)	
G4	1 (2.5)	0 (0)	
**ECOG**			0.755
0	23 (57.5)	15 (57.7)	
1	10 (25.0)	8 (30.8)	
2	7 (17.5)	3 (11.5)	
CA 19–9 [U/ml]	207 (32; 758)	330 (80; 2263)	0.194
CEA [ng/ml]	3.1 (2.0; 5.7)	2.9 (1.8; 27.0)	0.708
Total bilirubin [mg/dl]	1.8 (0.7; 5.4)	1.0 (0.5; 2.0)	0.061
gGT [U/l]	720 (313; 1188)	396 (210; 980)	0.358
Aspartate aminotransferase [U/l]	67 (44; 145)	49 (33; 88)	0.121
Alanine aminotransferase [U/l]	70 (37; 125)	81 (40; 120)	0.990
Alkaline phosphatase [U/l]	471 (258; 630)	385 (187; 266)	0.325
INR [U/l]	1.0 (1.0; 1.1)	1.0 (1.0; 1.0)	0.095
MELD score	10.1 (7.0; 13.8)	6.7 (6.4; 9.8)	**0.015**
Creatinine [mg/dl]	0.8 (0.7; 1.0)	0.7 (0.6; 0.9)	0.227
CRP [mg/l]	17.7 (9.8; 55.9)	14.1 (3.9; 35.9)	0.287
Neutrophiles [/nl]	6.0 (3.7; 8.6)	4.6 (3.4; 6.7)	0.069
Lymphocytes [/nl]	1.4 (1.0; 1.9)	1.3 (0.9; 1.8)	0.660
Blood neutrophil to lymphocyte ratio	4.8 (2.3; 7.5)	4.1 (2.6; 5.1)	0.454

Categorical data are presented as absolute frequency with relative frequency in parentheses. Numerical data are presented as median with under and upper quartile in parentheses.

P values of categorical data refer to Chi-squared test or Fisher exact test between groups eRFA + CT and CT. P values of numerical data refer to Student unpaired t test or Mann–Whitney test between groups eRFA + CT and CT.

*CA19-9* carbohydrate antigen 19-9, *CCA* cholangiocarcinoma, *CEA* carcinoembryonic antigen, *CRP* C-reactive protein, *ECOG* Eastern Cooperative Oncology Group performance status, *gGT* gamma-glutamyltransferase, *INR* international normalized ratio.

This study was approved by the Ethics Committee of the Medical Faculty of the University of Bonn (No. 341/17) and was conducted in accordance to the Declaration of Helsinki. Written, informed consent was obtained from the patients before therapy beginning.

### Statistical analysis

Normal distribution of continuous variables was tested with the Kolmogorov–Smirnov test. Differences in continuous variables, expressed as medians and first and third quartiles, were assessed using Student unpaired t test or non-parametric Mann–Whitney test, as appropriate. Categorical variables, expressed as absolute frequencies and percentages, were compared using Pearson’s Chi squared test or Fisher exact test, as appropriate. Survival was compared by log-rank test and transcribed into Kaplan–Meier diagrams. Survival is presented as median and with 95% confidence interval (CI). Univariate and multivariate analyses were performed using Cox regression forward conditional models. Parameters with p-values ≤ 0.1 in univariate analysis were included in multivariate analysis. Results are expressed as hazard ratio (HR) and 95% confidence interval. Two-tailed p-values ≤ 0.05 were considered statistically significant. SPSS version 22 (IBM Corporation, Armonk, NY, USA, https://www.ibm.com/products/spss-statistics) was used for statistical analysis.

## Results

### Baseline and therapy characteristics

Between 2010 and 2020, 66 patients fulfilled the inclusion criteria: 26 (39.4%) patients were treated with CT alone and 40 (60.6%) patients received a combined therapy with CT and concomitant eRFA. Baseline characteristics are shown in Table 1. Hilar CCA Bismuth types III and IV were the predominant tumor localization in both groups (77.5% for combination group and 73.1% for CT alone). Patients receiving eRFA + CT had a worse liver function determined by higher MELD score (p = 0.015) than patients receiving CT at time of diagnosis. There were no other significant baseline differences between the combination group and the CT alone group.

All patients treated with CT received either a combination of gemcitabine and platinum derivates (cisplatin, oxaliplatin) or gemcitabine monotherapy in first-line CT. There were no significant differences in protocols, number of received cycles of CT or applied second-line CT between the two groups.

During therapy, patients received bile duct stenting or percutaneous transhepatic cholangiography interventions (PTCD) at regular intervals. If feasible, eRFA was repeated every 3–4 months. However, the total number of ablation procedures varied considerably (1–21 procedures) due to clinical performance, progression of disease, and patient decision. Overall, we performed 126 eRFAs, 55% of all patients treated with eRFA received more than one ablation, while 12.5% received more than five procedures.

A total of 20 (30.3%) patients were treated with PDT at least once, with even distribution between the combination group and the CT alone group (p = 0.947).

Therapy characteristics are shown in Table 2.

**Table 2:** Therapy characteristics.

Parameters	eRFA + CT (n = 40)	CT (n = 26)	P-value
**Lines of chemotherapy**			0.627
Only first-line	31 (77.5)	22 (84.6)	
second-line or third-line	9 (22.5)	4 (15.4)	
**First-line protocol**			0.920
Gemcitabine/cisplatin	29 (72.5)	20 (76.9)	
Gemcitabine/oxaliplatin	2 (5.0)	1 (3.8)	
Gemcitabine mono	9 (22.5)	5 (19.2)	
**Second-line protocol**			0.165
FOLFIRI	4 (50.0)	1 (33.3)	
Cetuximab/pembrolizumab	3 (37.5)	0 (0)	
Capecitabine	1 (12.5)	2 (66.7)	
N of first-line chemotherapy cycles	5.5 (3.0; 10.3)	6 (2; 8.3)	0.680
N of eRFA	2 (1; 4)	–	–
**eRFA procedure**			–
Endoscopic approach	38 (95.0)	–	
Percutaneous approach	2 (5.0)	–	
Emergency ERC	11 (27.5)	8 (30.8)	0.774
**PTCD**			0.249
Primary	2 (5.0)	2 (7.7)	
After resection with alternated anatomy	1 (2.5)	8 (30.8)	
Disease progression	1 (2.5)	1 (3.8)	
SIRT	1 (2.5)	1 (3.8)	0.755
Photodynamic therapy	12 (30.0)	8 (30.8)	0.947
**Prior surgical therapy**			0.254
No surgery	19 (47.5)	10 (38.5)	
Curative intended resection with recurrence	5 (12.5)	7 (26.9)	
Exploration, but no curative surgery possible	13 (32.5)	9 (34.6)	
Metastatic surgery	3 (7.5)	0 (0)	

Categorical data are presented as absolute frequency with relative frequency in parentheses. Numerical data are presented as median with under and upper quartile in parentheses.

*ERC* endoscopic retrograde cholangiography, *eRFA* endobiliary radiofrequency ablation, *FOLFIRI* chemotherapy regimen including folinic acid, fluorouracil and irinotecan, *SIRT* selective internal radiation therapy.

P values of categorical data refer to Chi-squared test or Fisher exact test between groups eRFA + CT and CT. P values of numerical data refer to Student unpaired t test or Mann–Whitney test between groups eRFA + CT and CT.

### Analysis of survival

**Figure 2:**
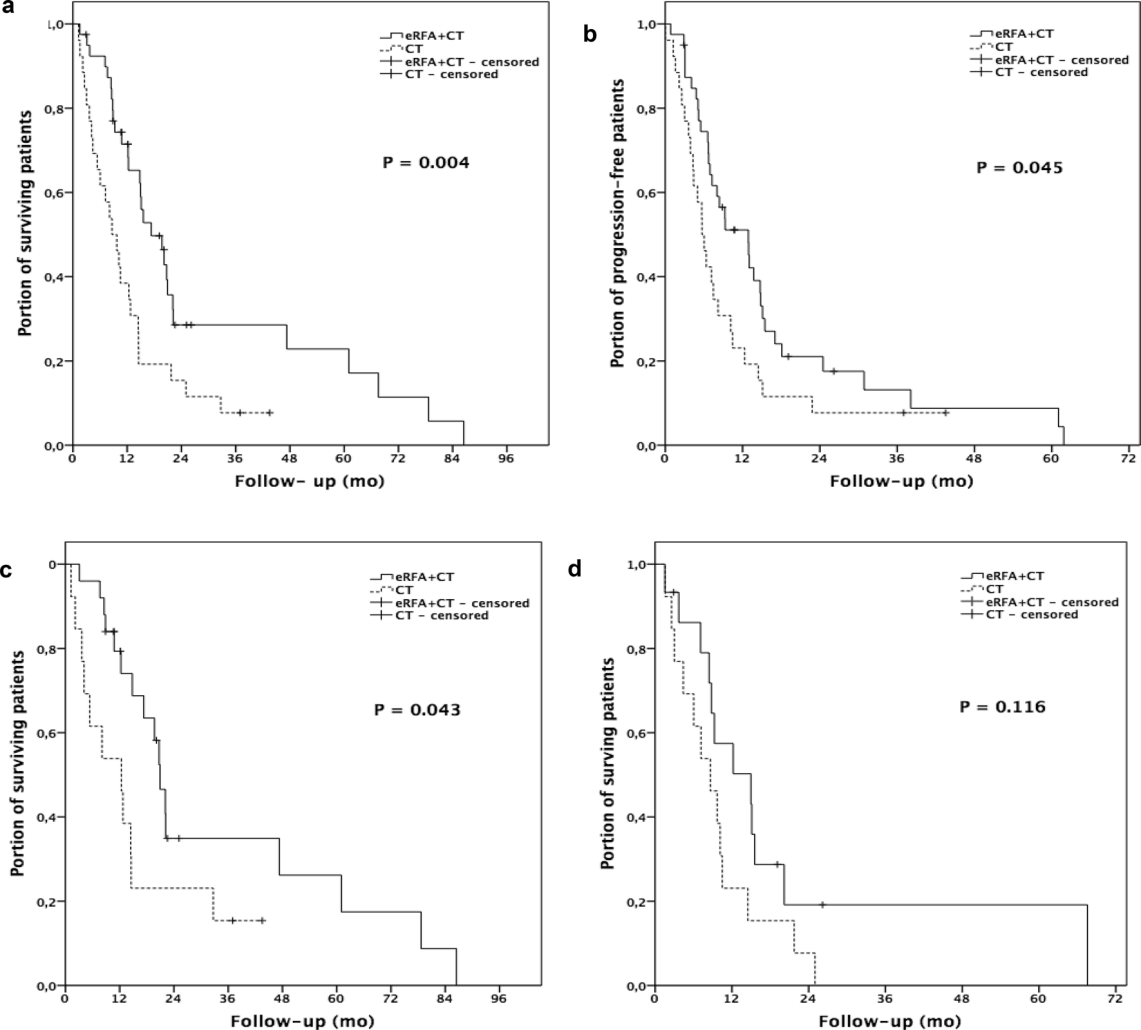
Kaplan–Meier survival analysis, with Log-Rank P. (**a**) Overall survival: eRFA + CT vs. CT alone. (**b**) Progression free survival: eRFA + CT vs. CT alone. (**c**) Overall survival of patients with non-metastatic disease: eRFA + CT vs. CT alone. (**d**) Overall survival of patients with metastatic disease: eRFA + CT vs. CT alone. *CT* chemotherapy, *eRFA* endobiliary radiofrequency ablation.

The median OS was 17.3 months (95% CI 10.9, 23.8) in the combination group and 8.6 months (95% CI 4.9, 12.4) in the CT alone group. (PFS) was 12.9 months (95% CI 7.8, 18.0) and 5.7 months (95% CI 4.0, 7.4) in the combination and the CT alone group, respectively. OS and PFS were significantly longer in the combined therapy group, determined by log-rank tests (p = 0.004 and p = 0.045, respectively). Kaplan‐Meier analysis of OS and PFS for the combination group vs. CT alone group is shown in Fig. 2a,b.

### Subgroup analysis

A subgroup analysis of patients with locally advanced disease vs. patients with metastatic disease revealed a survival benefit for the former when treated with combined CT + eRFA. Median OS was 20.9 months (95% CI 17.9, 24.0) for the combination group vs. 12.4 months for the CT alone group (95% CI: 3.7, 21.0) for non-metastatic disease and 15.0 months (95% CI 4.7, 25.3) vs. 8.6 months (95% CI 4.3, 13.0) for patients with metastatic disease. Comparison by log-rank test showed a significant survival benefit for the combination group in locally advanced stage (p = 0.043) that disappeared in the presence of extrahepatic metastases (p = 0.116), (Fig. 2c,d).

### Univariate and multivariate analysis

The parameters identified as significant predictors of survival by univariate analysis are shown in Table 3. In a multivariate Cox regression analysis, combined eRFA with CT (HR: 0.422, 95% CI 0.218, 0.816, p = 0.010) and initial surgery with tumor resection (HR: 0.201, 95% CI 0.068, 0.596, p = 0.004) remained significant independent predictors for survival.

**Table 3:** Univariate and multivariate time‐to‐event analysis of baseline and therapy characteristics.

Parameters	P-value	HR	HR 95% CI	
			Under	Upper
**Univariate analysis**				
Age	0.186	0.984	0.961	1.008
Female gender	0.593	0.857	0.485	1.512
Localization of tumor	0.127	1.595	0.875	2.904
M1	**0.019**	1.941	1.113	3.383
Histological grading	0.644	1.067	0.811	1.403
Albumin	**0.042**	0.958	0.919	0.998
CRP	0.130	1.006	0.998	1.015
gGT	0.081	1.000	1.000	1.001
Alanine aminotransferase	0.237	1.002	0.999	1.005
Aspartate aminotransferase	0.834	1.001	0.996	1.005
CA19-9	0.142	1.000	1.000	1.000
ECOG at diagnosis	0.051	1.436	0.999	2.063
MELD score	0.257	1.036	0.974	1.103
eRFA + CT	**0.005**	0.438	0.248	0.775
Photodynamic therapy	0.875	0.955	0.536	1.701
No surgery	0.256	1.378	0.792	2.398
Primary surgery with tumor resection	0.059	0.461	0.207	1.029
Primary surgery without tumor resection	0.967	0.988	0.558	1.750
PTCD	0.416	1.303	0.688	2.467
**Multivariate analysis**				
eRFA + CT	**0.010**	0.422	0.218	0.816
Primary surgery with tumor resection	**0.004**	0.201	0.068	0.596

*CA19-9* carbohydrate antigen 19-9, *CRP* C-reactive protein, *CT* chemotherapy, *ECOG* Eastern Cooperative Oncology Group performance status, *eRFA* endobiliary radiofrequency ablation, *gGT* gamma-glutamyltransferase, *MELD score* model of end stage liver disease score, *PTCD* percutaneous transhepatic bile duct drainage.

### Toxicity

Distribution of adverse events (AE) and toxicity is shown in Table 4. Cholangitis was the most frequently observed adverse event during therapy, with more episodes in the combination group (p = 0.031). Interestingly, there were no significant differences in the frequency of post-interventional cholangitis and other typical intervention-related complications, such as bleeding, pancreatitis, abscess or biloma formation between the combination group and the CT alone group. Hematological toxic effects occurred equally in both groups receiving CT. No further significant differences were found between the two groups.

**Table 4:** Adverse events.

Parameters	eRFA + CT (n = 40)	CT (n = 26)	P-value
Cholangitis during therapy	29 (72.5)	14 (53.8)	**0.031**
**ERCP associated**	11 (27.5)	9 (34.6)	0.539
Pancreatitis	4 (10.0)	2 (7.7)	0.750
Cholangitis	7 (17.5)	3 (11.5)	0.257
Biloma	1 (2.5)	0 (0)	0.417
Bleeding	3 (7.5)	4 (15.4)	0.420
Abscess	1 (2.5)	1 (3.8)	0.755
**eRFA associated**	6 (15.0)	–	–
Cholangitis	5 (12.5)	–	–
Abscess	1 (2.5)	–	–
**Hematologic toxic effects**			
Thrombocytopenia	9 (22.5)	10 (38.5)	0.162
Neutropenia	4 (10.0)	4 (15.4)	0.702
Anemia	10 (25.0)	11 (42.3)	0.140
Leucopenia	6 (15.0)	3 (11.5)	0.689
Nephrotoxicity	3 (7.5)	0 (0)	0.273
Fatigue	3 (7.5)	2 (7.7)	0.977
Thromboembolic event	3 (7.5)	5 (19.2)	0.247

Data are presented as absolute frequency with relative frequency in parentheses.

Adverse events were registered if they required intervention or adaptation of therapy (CTCAE v4.0, grades 3–5).

*ERCP* endoscopic retrograde cholangiopancreatography, *eRFA* endobiliary radiofrequency ablation.

P values refer to Chi-squared test or Fisher exact performed between groups eRFA + CT and CT.

## Discussion

In this retrospective study, we found that endobiliary RFA in combination with systemic CT was a feasible and safe treatment regimen in our cohort of patients with unresectable eCCA that was associated with a significantly prolonged median survival (17.3 vs. 8.6 months; p = 0.004) and PFS (12.9 vs. 5.7 months; p = 0.045) compared to current standard treatment with systemic CT alone.

Locally advanced or metastatic cholangiocarcinoma are difficult to manage and limited to palliative treatment options that aim to improve patient survival and quality of life. The current standard first-line treatment option for irresectable eCCA is systemic CT with gemcitabine ± platinum-based agents^8^.

However, the median OS is still less than one year in studies evaluating standard first-line CT, while in studies with second-line therapies, an OS up to 12.1 months has been reported^22,23^.

In addition to systemic treatment, advanced eCCA requires the endoscopic management of malignant bile duct strictures with the goal of optimal biliary drainage in order to avoid cholestasis and cholangitis. This can be done effectively through the implantation of biliary plastic or metal stents. To date, local ablative tumor therapy with PDT or eRFA has not been generally recommended for palliative treatment of eCCA. However, there is some evidence that these techniques could prolong stent patency and thus improve overall survival. In a recent retrospective study from our group, we found that PDT combined with CT resulted in significantly longer OS than CT alone^24^. However, phototoxicity of the photosensitizer is not acceptable for all patients, limiting the use of PDT. Furthermore, a laser is required for PDT, which is not available in all endoscopy units. In contrast, eRFA has no systemic side effects, since the effect of local, high temperatures is limited to the surrounding tissue and neither additional equipment nor specific drugs are needed.

Since Steel et al. reported on the use of eRFA for the treatment of malignant biliary obstruction in 2011, several further studies have demonstrated the safety and the improved maintenance of the bile duct system through eRFA and the influence of eRFA on survival of unresectable eCCA^17,18,21,25–29^(Table 5). However, all these studies focus on the efficacy of eRFA compared to stenting alone, disregarding the influence of current standard systemic CT, by excluding patients with CT or by matching controls with equal CT status. Accordingly, the safety and the efficacy of eRFA in combination with palliative CT for the treatment of unresectable eCCA remains unclear to date.

**Table 5:** Comparison of other publications on eRFA and CT in CCA.

Study	Design	Number of patients with eCCA	Comparison groups with patients with eCCA		P-value	Percentage of patients with concomitant CT
			eRFA ± CT	Stenting ± CT		
**eRFA + CT vs. CT**						
Gonzalez et al. (2021)	Retrospective vs. control	66	Median OS: 17.3	Median OS: 8.6	0.004	100
**eRFA vs. stenting**						
Sharaiha et al. (2014)^15^	Retrospective	37	Collectively median OS: 5.9		0.87	Not shown
Dolak et al. (2014)^16^	Retrospective single arm	51	Median OS: 10.9			39
Liang et al. (2015)^27^	Retrospective	76	Median OS: 12.7^a,c^	Median OS: 11.4^a^	0.036	67
Sharaiha et al. (2015)^17^	Retrospective	45	Mean OS: 17.7	Mean OS: 5.9	< 0.001	78^b,c^
Laquiere et al. (2016)^18^	Prospective	12	Mean OS: 12.3			25
Yang et al. (2018)^21^	RCT	65	Mean OS: 13.2	Mean OS: 8.3	< 0.001	0
Bokemeyer et al. (2019)^20^	Retrospective	42	Mean OS: 11.4	Mean OS 7.4	0.046	31
Kang et al. (2021)^28^	RCT	18	Median OS: 8.1	Median OS: 6	0.281	69^c^
Xia et al. (2021)^29^	Retrospective	335	Median OS: 11.3	Median OS: 6.9	< 0.001	4
Brandi et al. (2020)^32^	Retrospective	29^d^	Median OS for intrahepatic RFA: 27.5			34

Overall survival is presented in months. P-values refer to log rank test.

*CCA* cholangiocarcinoma, *CT* chemotherapy, *eRFA* endobiliary radiofrequency ablation, *OS* overall survival, *RCT* randomized controlled trial.

^a^Visually estimated median OS based on Kaplan–Meier survival curve.

^b^Only shown for eRFA-group.

^c^Only shown for all tumor localizations included in the study.

^d^Intrahepatic cholangiocarcinoma.

Consecutively, we aimed in our analysis to compare the outcome of additional combined eRFA with standard CT vs. standard CT alone. The median survival of the eRFA CT combination group (17.3 months), where the majority of patients had a Bismuth type III and IV hilar CCA, is slightly longer than most results of the already published studies (Table 5).

Furthermore, the combination therapy with eRFA and CT was a significant independent predictor of prolonged survival in the univariate as well as in the multivariate analysis, supporting the significant log-rank test result for OS for combination therapy vs. CT alone. These findings correspond to a combination of the results from Yang et al., whose multivariate analysis revealed eRFA as a main protecting factor improving patient survival, and from Sharaiha et al. and Liang et al., whose multivariate analysis presented CT as a significant predictor of improved survival^17,21,27^.

Contrary to our previous promising results, PDT was not associated with prolonged survival in this study^24^. However, with the availability of eRFA in our center, patients requested more eRFA for intraductal treatment of eCCA due to less side effects (phototoxicity). Hence, eRFA partly replaced PDT as first-line approach and PDT was only performed when eRFA failed, when it was technically impossible or when it was requested explicitly by the patient as first-line treatment. This kind of negative selection bias might explain the observed inefficacy of PDT. Prospective randomized studies comparing PDT and eRFA as treatment approaches for intraductal therapy of eRFA are urgently needed.

Compared to the results of the phase III ABC-02 trial, which reported a median survival of 11.7 months for gemcitabine and cisplatin, and the trial of Dierks et al., which reported a 9.5 and 9.6 months OS for their CT groups, our eRFA + CT combination group had a longer OS of 17.3, which we regard as promising data reinforcing the possible beneficial role of eRFA for patients with eCCA^8,30^.

The results of the present study provide evidence for the feasibility and tolerability of the combination of eRFA and CT, resulting in no relevant differences in frequency of hematologic toxic events compared to CT alone. Hence, no difference in dose adjustment of CT was observed. The pooled rate of adverse events after eRFA is reported with 17% (95% CI 10%, 25%)^31^. We found a comparable complication rate of 15% for the combination group. Analysis of cholangitis, the most common adverse event in CCA, showed a higher frequency of therapy-related cholangitis for the combination group compared to chemotherapy alone (p = 0.031). This might be explained by the fact that eRFA-induced necrotic tissue leads to the occlusion of biliary stents. Furthermore, a selection bias cannot be excluded for the combination group, in whose patients obstructive cholangitis is seen more often due to primary eRFA-indication-giving biliary obstruction. No differences were found concerning any ERCP-related complication (p = 0.539), which is somewhat surprising due to the significant difference in median applied ERC interventions in the combination group (eight interventions vs. three interventions, p < 0.001).

In agreement with Xia et al., our subgroup analysis revealed a significantly improved survival through combination therapy in non-metastatic eCCA (20.9 vs. 12.4 months, p = 0.043), while the effect disappeared in the presence of metastatic disease (15.0 vs. 8.6 months, p = 0.116)^29^. These findings suggest a benefit for the combination therapy in eCCA with non-metastatic status, but a reduced influence in patients with M1 status. Future studies are required to evaluate in more detail the systemic effect of eRFA, providing information on more precise selection criteria for treatment with eRFA in patients with unresectable eCCA.

The beneficial effect of eRFA for eCCA reported in this study is also in line with the increasing evidence reported for the use of local therapy (e.g., RFA) in the therapy of intrahepatic cholangiocarcinoma (iCCA). Brandi et al. made an interesting amendment for optimization for RFA effectiveness. Their retrospective study identified intrahepatic tumor lesions < 20 mm as an independent prognostic parameter for longer progression-free survival after percutaneous ultrasound-guided RFA. Additionally, the number of overall nodules treated with RFA as well as the sum of diameter of nodules at the moment of first RFA were significant parameters affecting overall survival^32^. Therefore, it might be reasonable to perform additive intrahepatic RFA in patients with intrahepatic lesions < 20 mm.

In summary, our study is limited by its retrospective single center design and as therapy decisions were made based on clinical judgement, a selection bias cannot be completely excluded. However, with a cumulative number of 125 eRFA treatments and 40 patients receiving eRFA, it displays one of the largest data records of eRFA for the therapy of unresectable eCCA. Furthermore, our two groups were well balanced in terms of baseline characteristics and our study is the first to show that eRFA in combination with systemic CT is a safe and beneficial treatment regimen for the heterogenous group of patients with unresectable and mainly hilar eCCA and that it can significantly prolong the OS compared to current standard treatment with systemic CT only. To provide a general recommendation for this promising treatment option in patients with eCCA, prospective randomized confirmatory studies are urgently needed.

## Data Availability

The data used and analyzed during the current study are available from the corresponding author on reasonable request.

## References

[1] Marcano-Bonilla L, Mohamed EA, Mounajjed T, Roberts LR (2016). Biliary tract cancers: epidemiology, molecular pathogenesis and genetic risk associations. Chin. Clin. Oncol..

[2] Walter D (2019). Cholangiocarcinoma in Germany: Epidemiologic trends and impact of misclassification. Liver Int..

[3] von Hahn T (2011). Epidemiological trends in incidence and mortality of hepatobiliary cancers in Germany. Scand. J. Gastroenterol..

[4] Doherty B, Nambudiri VE, Palmer WC (2017). Update on the diagnosis and treatment of cholangiocarcinoma. Curr. Gastroenterol. Rep..

[5] Primrose JN (2019). Capecitabine compared with observation in resected biliary tract cancer (BILCAP): A randomised, controlled, multicentre, phase 3 study. Lancet Oncol..

[6] Rizzo A, Brandi G (2021). BILCAP trial and adjuvant capecitabine in resectable biliary tract cancer: Reflections on a standard of care. Expert Rev. Gastroenterol. Hepatol..

[7] DeOliveira ML (2007). Cholangiocarcinoma: Thirty-one-year experience with 564 patients at a single institution. Ann. Surg..

[8] Valle J (2010). Cisplatin plus gemcitabine versus gemcitabine for biliary tract cancer. N. Engl. J. Med..

[9] Rizzo A, Brandi G (2021). First-line chemotherapy in advanced biliary tract cancer ten years after the ABC-02 trial: “and yet it moves!”. Cancer Treatm. Res. Commun..

[10] Abou-Alfa GK (2020). Pemigatinib for previously treated, locally advanced or metastatic cholangiocarcinoma: A multicentre, open-label, phase 2 study. Lancet Oncol..

[11] O'Brien S (2020). Comparing the efficacy of initial percutaneous transhepatic biliary drainage and endoscopic retrograde cholangiopancreatography with stenting for relief of biliary obstruction in unresectable cholangiocarcinoma. Surg. Endosc..

[12] Mensah ET, Martin J, Topazian M (2016). Radiofrequency ablation for biliary malignancies. Curr. Opin. Gastroenterol..

[13] Wadsworth CA, Westaby D, Khan SA (2013). Endoscopic radiofrequency ablation for cholangiocarcinoma. Curr. Opin. Gastroenterol..

[14] Kim GH (2021). Thermal ablation in the treatment of intrahepatic cholangiocarcinoma: A systematic review and meta-analysis. Eur. Radiol..

[15] Sharaiha RZ (2014). Comparison of metal stenting with radiofrequency ablation versus stenting alone for treating malignant biliary strictures: Is there an added benefit?. Dig. Dis. Sci..

[16] Dolak W (2014). Endoscopic radiofrequency ablation for malignant biliary obstruction: A nationwide retrospective study of 84 consecutive applications. Surg. Endosc..

[17] Sharaiha RZ (2015). Impact of radiofrequency ablation on malignant biliary strictures: results of a collaborative registry. Dig. Dis. Sci..

[18] Laquiere A (2016). Safety and feasibility of endoscopic biliary radiofrequency ablation treatment of extrahepatic cholangiocarcinoma. Surg. Endosc..

[19] Wang Y (2016). Percutaneous intraductal radiofrequency ablation in the management of unresectable Bismuth types III and IV hilar cholangiocarcinoma. Oncotarget.

[20] Bokemeyer A (2019). Endoscopic radiofrequency ablation prolongs survival of patients with unresectable hilar cholangiocellular carcinoma: A case-control study. Sci. Rep..

[21] Yang J (2018). Efficacy and safety of endoscopic radiofrequency ablation for unresectable extrahepatic cholangiocarcinoma: A randomized trial. Endoscopy.

[22] Moik F (2019). Benefit of second-line systemic chemotherapy for advanced biliary tract cancer: A propensity score analysis. Sci. Rep..

[23] Mizrahi JD (2020). Multi-institutional retrospective analysis of FOLFIRI in patients with advanced biliary tract cancers. World J. Gastrointest. Oncol..

[24] Gonzalez-Carmona MA (2019). Combined photodynamic therapy with systemic chemotherapy for unresectable cholangiocarcinoma. Aliment Pharmacol. Ther..

[25] Steel AW (2011). Endoscopically applied radiofrequency ablation appears to be safe in the treatment of malignant biliary obstruction. Gastrointest. Endosc..

[26] Figueroa-Barojas P (2013). Safety and efficacy of radiofrequency ablation in the management of unresectable bile duct and pancreatic cancer: A novel palliation technique. J. Oncol..

[27] Liang, H., Peng, Z., Cao, L., Qian, S. & Shao, Z. Metal stenting with or without endobiliary radiofrequency ablation for unresectable extrahepatic cholangiocarcinoma. J. Cancer Ther. 6(11), 12. 10.4236/jct.2015.611106 (2015).

[28] Kang H (2021). Efficacy and safety of palliative endobiliary radiofrequency ablation using a novel temperature-controlled catheter for malignant biliary stricture: A single-center prospective randomized phase II TRIAL. Surg. Endosc..

[29] Xia MX (2021). Effect of endoscopic radiofrequency ablation on the survival of patients with inoperable malignant biliary strictures: A large cohort study. J. Hepatobiliary Pancreat. Sci..

[30] Dierks J (2018). Translating the ABC-02 trial into daily practice: outcome of palliative treatment in patients with unresectable biliary tract cancer treated with gemcitabine and cisplatin. Acta Oncol.

[31] Zheng X (2016). Endoscopic radiofrequency ablation may be preferable in the management of malignant biliary obstruction: A systematic review and meta-analysis. J. Dig. Dis..

[32] Brandi G (2020). Percutaneous radiofrequency ablation in intrahepatic cholangiocarcinoma: a retrospective single-center experience. Int. J. Hyperth..

